# Erratum

**DOI:** 10.1155/S1463924680000466

**Published:** 1980

**Authors:** Michael A. Koupparis, Ken M. Walczak, Howard V. Malmstadt


### Error messages

The error detection system in the C analyser adds validity to the answers. A series of messages are used which indicate whether or not there are errors in calibration or in the reaction temperature, that the initial absorbance of the kinetic reaction is too high or too low, that the reaction rate curves fail to satisfy the linearity criteria etc. However, the linearity error code is printed only if the linearity error is greater than 20%. The acceptability limits of linearity should be smaller and/or the operator should be able to select a value for them. The graphical print-out of kinetic measurements is not possible in the C analyser, as it is in the System Olli 3000 analyser. However, if the error message indicates the nonlinear reaction, the user can check how the reaction is proceeding by using RATE DATA key. Rate data consists of (1) four abosrbance values, which are mean values of three successive readings, (2) three dA/min values obtained from the successive mean absorbances respectively, and (3) results in concentration units, which are calculated for each dA/min. The error messages are not included in the programs of the System Olli 3000 analyser.

### Reliability

The primary purpose of this evaluation was to test the suitability of the C Parallel Analyser for the laboratories in the district of Oulu University Central Hospital. During the two months of the evaluation period no service was needed. After the analyser had been in Oulu City Hospital for one year, the downtime was three days, one due to the change of the UV-filter, and two due to the repair of the ignition unit for the Xenon-lamp. Additionally, the timer of the preincubator was broken, but it did not hold up work. In Oulu City Hospital the analyser is used for five days weekly and about 300 analyses are performed daily.

### Conclusion

The C Parallel Analyser has proved to be an instrument with good reliability and precision. Advantages were the speed of analysis, the ability to change chemistries easily and quickly, versatility in accommodating kinetic and equilibrium tests equally well, and the ease with which methods can be modified. The ease of operation is impressive and the operator can learn the keyboard manipulations rapidly. Errors and malfunctions are indicated through a thermal printer. Because standards and samples can be analysed in parallel, the C analyser has much potential especially in the kinetic analyses of serum nonenzymatic constituents. However, because the first readings can be made only within 30 seconds after initiating the reaction, analyses of very rapid kinetic reactions, for instance kinetic turbidimetric assays of serum immunoglobulins are difficult. The C analyser also lacks a graphic printer.

A semi-automatic diluter (or the D parallel sample processor) is needed for the pipetting of small sample volumes. The analyser should have an application in performing a range of analyses in laboratories where around 150-600 daily analyses are performed and it is also suitable for the analysis of emergency samples.

ACKNOWLEDGEMENTS

The loan of the analyser used in this investigation was arranged by Kone Oy, Instrument Division. Marjatta Leppilampi, MSc, is thanked for excellent technical assistance and Maija-Leena Kallio, MSc, for providing information on the reliability of the instrument.

REFERENCES

[1] Adlercreutz, H., Peltonen, V., and Voipio, T., *Clinical Chemistry,* 1975, **21**, 676.

[2] Puukka, R. and Puukka, M. Third European Congress of Clinical Chemistry, Brighton, England, June 3-8, *Abstract book,* 1979, p. 44.

[3] Committee on Enzymes of the Scandinavian Society for Clinical Chemistry and Clinical Physiology. *Scandinavian Journal of Clinical Laboratory Investigations,* 1974, **33**, 291.

[4] Puukka, R. *Scandinavian Journal of Clinical Laboratory Investigations,* 1978, **38**, 127.

[5] Puukka, R., Jokela, H. and Puukka, M. *Scandinavian Journal of Clinical Laboratory Investigations,* 1978, **38**, 189.

[6] Hammond, G.L., Leinonen, P. and Vihko, R. *Clinical Chemistry,* 1979, **25**, 129.

[7] Spencer, K. and Price, C.P. *Annals of Clinical Biochemistry,* 1977, **14**, 105.

[8] Cederblad, G., Hickey, B.E., Hollender, A., and Åkerlund, G. *Clinical Chemistry,* 1978, **24**, 1191.

### Erratum

*The Journal of Automatic Chemistry,* April 1980, **2**, **2**, 66-75.

**A compact automated microprocessor-based flow analyser**

**Michael A. Koupparis, Ken M. Walczak and Howard V. Malmstadt**

The authors have asked that the following errors in the above paper be brought to the attention of the readers.

On p71, the second sentence of the fourth paragraph should read, "The 1 to 0 transition was chosen as the power-on state of the parallel I/O ports to ensure that the control signals were not activated when power was applied to the microcomputer or a reset sequence was initiated."

On p71, the final paragraph should state, "The absorbance measurements of Table 2 were carried out at 520 mm."

On p73, in the first complete paragraph, the sixth and seventh sentences should read, "The minimum volume required to move the old solution from the cell (dead volume of mixer, flow cell, and their connections) was found to be 100 µl. 100-25 µl can be chosen by adjusting the mechanical stoppers of the automatic pipetter."

On p74, the ascorbic acid standards of the text and in Table 5 were made of 5-40 mg/l in 0.05M oxalic acid solution.

On p75, in the table of References, the fifth reference was published in 1977.

On p74, the slope in Table 5 should read −0.02366.

